# Endophyte diversity and its correlation with naphthoquinone metabolites in cultivated *Arnebia euchroma* across different growth years

**DOI:** 10.1371/journal.pone.0348171

**Published:** 2026-05-21

**Authors:** Jinrong Zhao, Jingjing Chen, Xuejia Zhang, Yanjiao Wang, Haiyan Xu

**Affiliations:** 1 Traditional Chinese medical science academy, Xinjiang Medical University, Xinjiang, China; 2 Xinjiang Key Laboratory of Famous Prescription and Science of Formulas, Xinjiang Medical University, Xinjiang, China; 3 Xinjiang Key Laboratory of Planting Standards for Authentic and Superior Chinese Medicinal Materials, Xinjiang Medical University, Xinjiang, China; 4 Institute of Traditional Chinese Medicine, Xinjiang Medical University, Xinjiang, China; 5 Department of Basic Medical Sciences, Xinjiang Medical University, Xinjiang, China; Sultan Qaboos University College of Science, OMAN

## Abstract

Previous studies on *Arnebia euchroma* (Royle) Johnst. have focused on its physicochemical and pharmacological properties, and studies on the differences in metabolites in *A. euchroma* have also highlighted the effects of external environmental factors. In this study, we compared the differences in the diversity of endophytes in the root tissue of *A. euchroma* grown for 1–4 years in Hejing County using high-throughput sequencing and analyzed their relationship with eight secondary metabolites, including alkannin, *β*,*β*-dimethylacrylshikonin, deoxyshikonin, acetylshikonin, *β*-acetoxyisovalerylshikonin,*β*-hydroxyisovalerylshikonin, isobutyrylshikonin, and isovalerylshikonin. The results indicate that a total of 1161 amplicon sequence variants (ASVs) of endophytic fungi from cultivated *A. euchroma* were classified into 12 phyla, 32 classes, 76 orders, 153 families, 259 genera, and 367 species. Additionally, 673 amplicon sequence variants (ASVs) of endophytic bacteria were classified into 18 phyla, 37 classes, 89 orders, 139 families, 226 genera, and 267 species. The α-diversity of endophytic fungi and bacteria in cultivated *A. euchroma* peaked in four-year-old (X-4Z) and two-year-old (X-2Z) respectively. Spearman’s correlation analysis showed that *β*-hydroxyisovalerylshikonin, acetylshikonin, isobutyrylshikonin, *β,β*-dimethylacrylshikonin, and isovalerylshikonin were correlated with endophytic fungal diversity and abundance (*P ≤ 0.05*). FUNGuild and PICRUSt2 predictive analyses showed that the dominant trophic modes of endophytic fungi in all samples were saprotrophic and pathotrophic, with the highest relative abundance in X-4Z, while the main trophic mode of endophytic bacteria was metabolic. Our results help to elucidate the importance of plant-microbe interactions and provide key information on the role of endophytes in promoting the cultivation of *A. euchroma* and the accumulation of its important secondary metabolites. They also provide a reference for further research on the effects of differences in the community composition of endophytes on the accumulation of medicinal compounds in *A. euchroma* and for the development of resources for its cultivation.

## Introduction

*Arnebia euchroma* (Royle) Johnst. is cold, sweet, and salty and enters the heart and liver meridians. It is a medicinal plant mostly harvested for its roots that produce naphthoquinone compounds such as shikonin, phenolic compounds, and their derivatives [1], which have a variety of pharmacological activities such as anti-inflammatory, antimicrobial, antitumor, and wound healing [2]. Currently, the main biologically active constituents of *A. euchroma* are alkannin, *β,β*-dimethylacrylshikonin, *β,β*-dimethylacrylshikoni, deoxyshikonin, acetylshikonin, *β*-acetoxyisovaleryshikonin, hydroxyisovalerylshikonin, isobutyrylshikonin and isovalerylshikonin.Numerous studies have shown that endophytes may be involved in the accumulation of secondary metabolites in host plants [3–8]. Xinjiang Uygur Autonomous Region (XUAR), is the local origin of *A. euchroma,* whose wild resources are on the verge of depletion due to the increase in wild excavation in recent years. Wild *A. euchroma* seeds have a low germination rate due to the high demand for external factors, which make wild resources increasingly scarce [9,10]. Therefore, the production of *A. euchroma* through asexual propagation has been increased. Because of its rich resources and a special ecological environment in HeJing County, a breeding base of *A. euchroma* has been established in this region. Although the production of *A. euchroma* has achieved some degree of success, it still faces some problems such as slow plant growth and poor quality. Therefore, to improve the quality of cultivated *A. euchroma,* we investigated the effects of differences in endophytic fungal and bacterial communities that inhabit the cultivated *A. euchroma* resources on the accumulation of its biologically active compounds. This paper aims to analyze the correlation between the diversity of endophytes and secondary metabolites in *A. euchroma* cultivated for 1–4 years through plant-microbe symbiosis.

Plant endophytes are microorganisms, including bacteria, fungi, and actinomycetes, that reside within tissues or intercellular spaces of a healthy plant during the majority of its life cycle, without causing the symptoms of infection [11]. Endophytes play important roles in gene expression, growth and development, metabolic pathways, and response to biotic stresses in plants [12,13]. For example, studies on *A. thaliana*, soybean, tomato, garlic, and *Salvia miltiorrhiza* have demonstrated their ability to increase antibiotic production and biofilm formation through plant-microbe interactions [14–16]. Recently, the concept of plant-microbe interactions and their associated genes has attracted much attention in terms of plant survival and adaptation [17]. Plants have co-evolved with their microbial symbionts, which are considered to play an important role in their life cycle. Since endophytes can be present in the tissues of host plants throughout their life cycle, they affect their cells directly or indirectly [18,19]. The symbiotic relationship between endophytes and host plants is maintained by inducing the production of metabolites required to promote plant growth or to protect it from pathogens and adverse environmental conditions [20], and therefore it is of great interest to researchers. Endophytes are closely associated with the primary and secondary metabolites [21,22] and their precursors in host plants [23]. Cui et al [24,25] found that the accumulation of secondary metabolites in licorice root was closely related to the composition of the endophytic fungal communities. Song et al. [26] observed that *Bacillus altitudinis*, an endophytic bacillus isolated from ginseng, enhanced the accumulation of ginsenosides, and Stierle et al. [27] found for the first time that the endophytic fungus isolated from redwood could produce paclitaxel, a medicinal ingredient with anticancer properties. Dang et al. [28] reported that during the plant’s growth, the content of three secondary metabolites in licorice root increased each year, and the endophytic fungal communities were more sensitive to these metabolites. Therefore, endophytic fungi play an important role in promoting plant growth and the accumulation of metabolites and screening the substitutes for medicinal plants.

High-throughput sequencing (HTS), a second-generation sequencing technology [29], not only comprehensively and objectively determines the structure and the relative composition of microbial communities in target environments but also contributes to the study of microbial diversity, compared to traditional culture methods [30,31]. HTS has been widely used in diversity analysis of symbiotic microbes [32], molecular labeling technology [33], disease risk assessment [34] and environmental testing [35]. In recent years, HTS has been used to predict the diversity and function of symbiotic microbes in Dendrobium [36], *Panax ginseng* [37], *Astragali Radix* [6], *Rheum palmatum* [38], *Panax quinquefolius L.* [39], and other plants. Furthermore, researchers employed HTS to analyze the diversity of cultivable endophytes in *A. euchroma* in different regions and hypothesized that in different organs, they are equally diverse and ecologically functional. In this study, for the first time, we focused on the root tissue of *A. euchroma* cultivated for 1–4 years to explain the differences in quality between cultivated and wild *A. euchroma* from a new perspective of endophytes. The main objectives of the present study were as follows: (1) to elucidate the diversity of endophytic fungi and bacteria in the root tissue of *A. euchroma* grown for 1–4 years; (2) to evaluate the correlation between the abundance of endophytic fungi and bacteria and the metabolites in the root tissue of *A. euchroma* cultivated for 1–4 years; (3) To perform the functional prediction of endophytic fungal and bacterial communities in roots of *A. euchroma* and analysis of their composition and diversity; (4) to perform the correlation analysis of root endophytes and eight secondary metabolites in *A. euchroma* using R programming language’s heatmap package. The results lay a theoretical foundation for further understanding of the plant-microbe relationship in *A. euchroma* and are of great significance for the cultivation and development of *Arnebia Radix* resources in Xinjiang and the improvement of their quality.

## Materials and methods

### Experimental materials

To compare the effects of different age treatments (1–4 cultivation years) on the diversity of root endophytes of *A. euchroma*, cultivated *A. euchroma* plants collected from the cultivation site in Hejing County, Bayin’guoleng Mongol Autonomous Prefecture, Xinjiang Uygur Autonomous Region (42°42’20.68″N, 84°06’28.69″E) were selected as plant materials. All plants were identified as *A. euchroma*. by Prof. Haiyan Xu, Xinjiang Medical University (voucher numbers: XJMU2023091003). Five biological replicates were performed for each age group. The collected fresh, healthy *A. euchroma* samples were labeled, photographed, and grouped according to the age treatment. The samples were then rinsed with distilled water to remove surface impurities, immersed in 75% ethanol for 5 minutes and 1% sodium hypochlorite for 3 minutes, and subsequently washed three times with sterile water. Finally, the sterile water from the final wash was spread onto potato dextrose agar (PDA) plates and nutrient agar (NA) plates, which were incubated at 28°C for 10 days and at 37°C for 5 days, respectively. The results of surface sterilization were observed to ensure that the DNA subsequently extracted originated solely from the interior of the root tissues [40,41]. Root samples of *A. euchroma* were allowed to dry on sterile filter paper and stored in EP tubes at –80 °C for subsequent high-throughput sequencing of endophytic fungi and bacteria in *A. euchroma*. All root samples were then divided into two parts, one was used for the analysis of endophyte diversity and another for the determination of the contents of secondary metabolites.

### DNA extraction, PCR amplification and high-throughput Sequencing

DNA extraction from *A. euchroma* was performed using Fast DNA® SPIN Kit (MP Bio, Santa Ana, USA). DNA amplification from *A. euchroma* samples was performed using the ABI GeneAmp® Model 9700 PCR instrument. The ITS1 region of fungal rDNA was amplified using the fungal-specific primers ITS1F (5’-CTTGGTCATTTAGAGGAAGTAA-3’) and ITS2R(5’-GCTGCGTTCTTCATCGATGC-3’). The 20 µL reaction system consisted of 2 µL of 10 × buffer, 2 µL of 2.5 mM dNTPs, 5 µmol/L of forward primer, 0.8 µL of reverse primer, 0.2 µL of TaKaRa Taq DNA polymerase, 0.2 µL of BSA, 10 ng of DNA template, and 20 µL ddH_2_O. The PCR program was run as follows: an initial denaturation at 95 ^◦^C for 3 min, denaturation at 95 ^◦^C for 30 s, annealing at 55 ^◦^C for 30 s, extension at 72 ^◦^C for 45 s, with a total of 35 cycles, and a final extension at 72 ^◦^C for 10 min, followed by the storage of samples at 10 ^◦^C. PCR products were detected by 2% agarose gel electrophoresis. Bacterial primers 338F (5’-ACTCCTACGGGGAGGCAGCA-3’) and 806R (5’-GGACTACHVGGTWTCTAAT-3’) were used to amplify the bacterial 16S rRNA gene (the V3-V4 region) [42]. The 20 µL reaction system consisted of 4 µL of 5 × buffer, 2 µL of 2.5 mM dNTPs, 0.8 µL (5 µM) of each primer, 0.4 µL of FastPfu DNA polymerase, 10 ng of DNA template, and 10 µL of ddH_2_O. PCR program was set as follows: an initial denaturation at 95 ^◦^C for 3 min, denaturation during cycling at 95 ^◦^C for 30 s, annealing at 52 ^◦^C for 30 s, extension at 72 ^◦^C for 45 s for a total number of 35 cycles, and a final extension at 72 ^◦^C for 5 min, followed by the storage at 10 ^◦^C [43]. Thereafter, PCR products were detected similarly by the 2% agarose gel electrophoresis. PCR amplification of the 16S rRNA gene and Illumina MiSeq sequencing were performed at Shanghai Majorbio Co., Ltd (Shanghai, China), with the project number MJ20230614095. Successful PCR products for all samples were pooled and purified using the EasyPureTM PCR Cleanup/Gel Extraction Kit (Axygen Biosciences, Union City, CA, USA). All samples were amplified thrice and pooled before sequencing. Purified PCR products were then sequenced on the Illumina NovaSeq platform [44].

### Quantitative analysis of metabolites in *A. euchroma*

A portion of the remaining dried roots of *A. euchroma* from the sample that was surface sterilized for DNA extraction was crushed and passed through a 60-mesh sieve. The contents of eight naphthoquinone components, including alkannin, *β,β*-dimethylacrylshikonin, deoxyshIkoninin, acetylshikonin, *β*-acetoxyisovalerylshikonin, *β*-hydroxyisovalerylshikonin, isobutyrylshikonin and isovalerylshikonin (purity ≥ 98%; Shanghai Yuanye Bio-Technology Co., Ltd., Shanghai, China) were determined by high-performance liquid chromatography (HPLC) using the methods described in the Chinese Pharmacopoeia 2020 edition [45,46]. Accordingly, 0.5 g of the powdered root of *A. euchroma* was weighed, placed in a conical flask, 25 mL of petroleum ether was added, and then the solution was allowed to be completely infiltrated. The sample was thereafter subjected to ultrasonic vibrations for 30 min, allowed to cool to make up for the loss of weight, and then filtered. Then, 10 mL of the filtrate was placed in an evaporating dish on a boiling water bath at 55 °C, dissolved in HPLC grade acetonitrile, and finally transferred into a 10 mL volumetric flask and diluted with HPLC acetonitrile to obtain the test solution. The contents of eight naphthoquinone components were determined on an Agilent 1260 high-performance liquid chromatography (HPLC) with an Agilent-C18 column (4.6 × 250 mm, 5 µm). The mobile phase was acetonitrile with 0.05% formic acid in water (70: 30), at a flow rate of 1 mL/min, a column temperature of 30 °C, a detection wavelength of 275 nm, and an injection volume of 10 µL [47,48]. The contents of eight naphthoquinones in all samples were determined using the standard curves obtained from the preliminary data of treatment groups.

### Data analysis

We performed alignment using the species annotation databases (UNITE 8.0/its_fungi and Silva 138/16s_bacteria). Taxonomic annotation was conducted using classify-sklearn with a confidence threshold of 0.7. Sequences were denoised using DADA2 (https://qiime2.org), and ASVs aligned to chloroplast and mitochondrial sequences were removed. The data were rarefied to the minimum sample depth, resulting in amplicon sequence variants (ASVs) with single-base accuracy [49]. The Mothur software v.1.30.2 was used to calculate the alpha diversity indices of the sample. The differences in alpha diversity indices of fungal and bacterial communities in *A. euchroma* between different age treatments were investigated using the Kruskal-Walli rank sum test. PCoA plots were generated using R software (version 3.3.1) on the Majorbio I-Sanger cloud platform. The ASV table was normalized using the weighted Bray-Curtis statistical algorithm, and *β*-diversity was evaluated using QIIME2 [50,51]. To compare the differences in endophytic fungal and bacterial community composition of *A. euchroma* grown for different years, the endophytic bacterial compositions at the phylum and genus levels were visualized and analyzed using R software (3.3.1) on the Majorbio I-Sanger Cloud Platform. Based on the relative abundance of endophytes, the number of common and endemic genera of endophytes was counted, and Venn diagrams were then constructed for *A. euchroma* samples from each age treatment. Linear discriminant analysis (LDA) effect size (LEfSe) identified taxa with the LDA scores greater than 2 as biomarkers, demonstrating the most differently abundant identified taxa between groups and the effect size of each significantly different taxon [52]. The FUNGuild (http://www.funguild.org/) and PICRUSt2 (http://huttenhower.sph.harvard.edu/galaxy) databases were used to predict the ecological functions of endophytic fungi and bacteria, respectively [53,54]. All statistical tests were performed using the analysis of variance (ANOVA) and Kruskal-Walli rank sum test, and the p-value of 0.01 was considered as the maximum significant level (*p < 0.01*), indicating a statistically significant result.

## Results

### The efficiency of surface sterilization

After incubation, no colonies appeared on both PDA and NA media, indicating that the sterilization of the sample was effective, and therefore, it could be used for subsequent experiments.

### Analysis of high-throughput sequencing data and alpha Diversity

**Fig 1:**
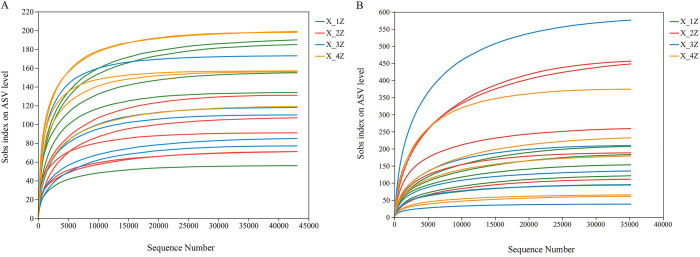
Dilution curves of endophytic fungi (A) and bacteria (B) in *A. euchroma* cultivated for different years.

**Fig 2:**
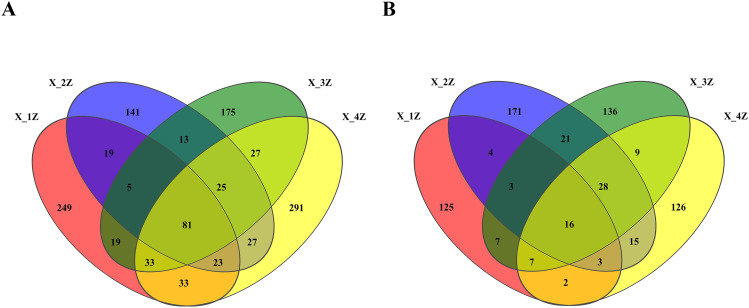
Venn diagrams of (A) endophytic fungi and (B) bacteria in *A. euchroma* cultivated for different years.

Twenty samples of *A. euchroma* cultivated for 1–4 years were subjected to HTS. After quality filtering and screening, a total of 43,114 high-quality sequences of endophytic fungi were obtained, with an average sequence length of 253 bp, and 1161 fungal ASVs were distributed with 12 phyla, 32 classes, 76 orders, 153 families, 259 genera, and 367 species. There were 35,232 high-quality sequences of endosymbiotic bacteria, with an average sequence length of 408 bp, and 673 bacterial ASVs were placed within 18 phyla, 37 classes, 89 orders, 139 families, 226 genera, and 267 species. The sparse curve can reflect the changes in species diversity and richness with sequencing volume. With the increase in sequencing volume, the sparse curve of the samples tended to stabilize, indicating that the amount of sequencing data tended to gradually become reasonable (Fig 1). Alpha diversity indices (Shannon’s, Chao, and Coverage) of *A. euchroma* grown for 1–4 years differed significantly among the groups, with the Shannon’s diversity index values being significantly different for fungal and bacterial communities. Moreover, this index followed the trends in the order of X-4Z > X-1Z > X-3Z > X-2Z and X-2Z > X-3Z > X-4Z > X-1Z, while the order of the Chao diversity index trends was X-4Z > X-1Z > X-3Z > X-2Z and X-2Z > X-3Z > X-4Z > X-1Z for fungi and bacteria, respectively. The coverage index reflects the community coverage, and the closer the value of the index to 1, the higher the coverage of the sample, the higher the probability of the detection of sequences in the sample, and the higher the accuracy of sequencing (Table 1). A total of 1161 fungal ITS ASVs and 673 bacterial 16S ASVs were identified in root samples collected from different years. The number of unique fungal ITS ASVs in 1–4year root samples was 249, 141, 175, and 291, respectively, while the number of unique bacterial 16S ASVs was 125, 171, 136, and 126, respectively (Fig 2).

**Table 1:** Diversity of fungal and bacterial communities.

Sample	Endophytic Fungi			Endophytic Bacteria		
	Shannon	Chao	Goods-Coverage	Shannon	Chao	Goods-Coverage
X-1Z	2.327	143.938	1.000	2.512	70.335	0.900
X-2Z	1.636	94.210	1.000	3.573	118.130	0.883
X-3Z	1.781	112.992	1.000	3.192	100.396	0.863
X-4Z	2.772	165.400	1.000	3.132	95.200	0.828

### Composition and relative abundance of endophytic bacterial and fungal communities in *A. euchroma* cultivated for different years

**Fig 3:**
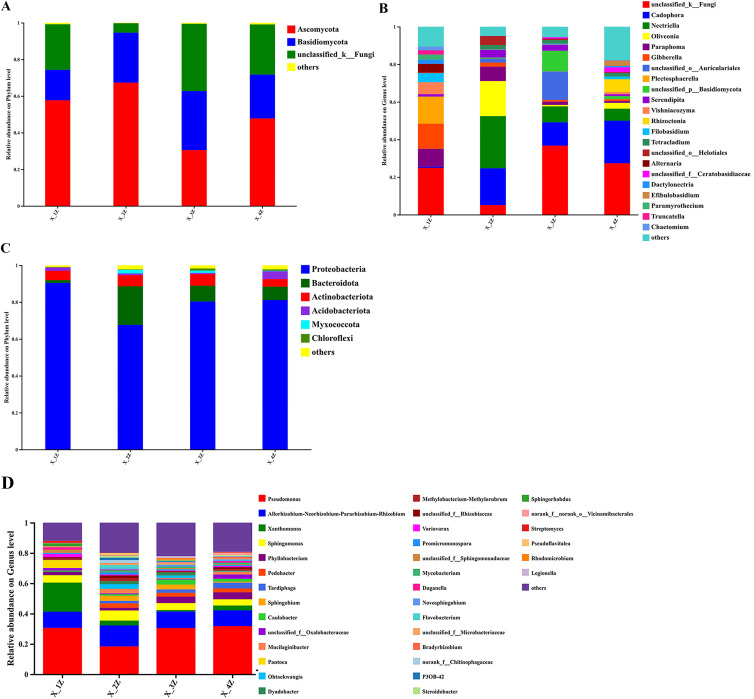
The relative abundance in root samples of *A. euchroma* grown for different years;(A) Endophytic fungal phyla; (B) Endophytic fungal genera; (C) Endophytic bacterial phyla; (D) Endophytic bacterial genera.

The ASVs from endophytic fungi in the root samples of *A. euchroma* grown for 1–4 years were classified into 12 phyla and 259 genera. At the phylum level, *Ascomycota* dominated fungal communities, with relative abundances ranging from 30.51% to 67.34% (Fig 3A). At the genus level, *Paraphoma*, *Gibberella*, and *Plectosphaerella* were the dominant fungal genera in the 1-year samples; *Cadophora* and *Nectriella* were the dominant fungal genera in the 2, 3, and 4-year samples. Additionally, *Oliveonia* was the dominant fungal genus in the 2-year samples (Fig 3B). The endophytic bacterial ASVs in root samples of *A. euchroma* grown for 1–4 years were classified into 18 phyla and 226 genera. At the phylum level, *Proteobacteria* dominated the bacterial community, with relative abundances ranging from 67.65% to 90.43% (Fig 3C). At the genus level, however, *Pseudomonas* was found as the dominant bacteria (18.37% ~ 31.77%) in root samples of *A. euchroma* grown for different years (Fig 3D).

### Beta diversity of endophytes

**Fig 4:**
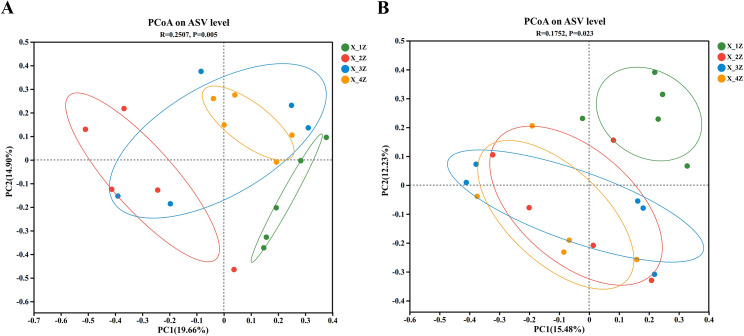
Principal coordinate analysis (PCoA) based on the relative abundance of (A) endophytic fungal and (B) endophytic bacterial ASVs in root samples of *A. euchroma* grown for different years.

The diversity of endophytes in *A. euchroma* cultivated for 1–4 years was assessed at the ASV level. Principal coordinate analysis (PCoA), based on the Bray-Curtis distance algorithm [55,56], and analysis of similarities (ANOSIM) were performed to test the inter-group differences. PC1 accounted for 19.66% of the total variation in the diversity of endophytic fungi, while PC2 accounted for 14.90% of it, with a high degree of dispersion within the fungal communities in *A. euchroma* for four groups of treatments and significant differences in the diversity of endophytic fungi in *A. euchroma* at different loci (*R = 0.2507, p = 0.0050*) (Fig 4A). The PC1 and PC2 could explain 15.48% and 12.23% of the total variation in diversity of endophytic bacteria, respectively, with a high degree of overlap among the bacterial communities in *A. euchroma* for three treatment groups (2–4years), and significant differences in beta diversity among the endophytic bacteria in *A. euchroma* at different loci (*R = 0.1752, p = 0.023*) (Fig 4B).

### Analysis of endophytic biomarkers in *A. euchroma*

**Fig 5:**
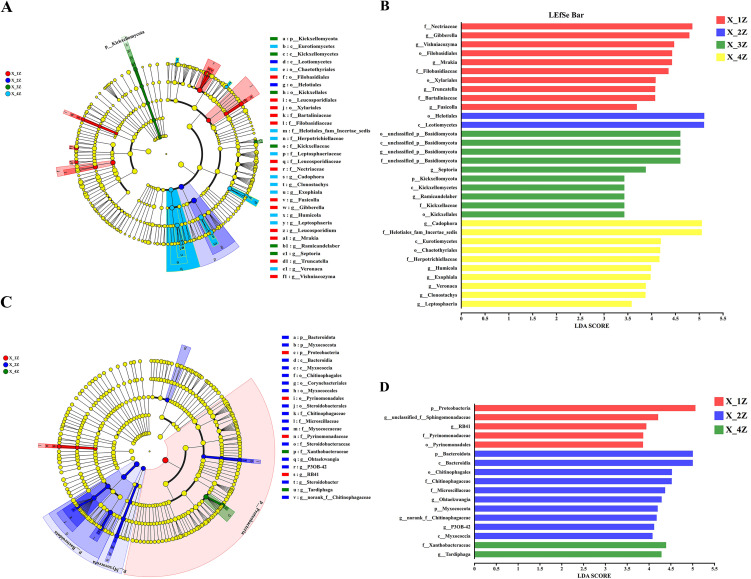
Biomarkers of endophytic bacteria and fungi in *A. euchroma* cultivated for different years; (A) Differential richness of endophytic fungal taxa in *A. euchroma* cultivated for different years was identified using the LEfSe analysis, and the taxa are listed on the right side of the pedigree plot. **(B)** Bar chart showing LDA scores for endophytic fungal taxa in root samples of *A. euchroma* grown for different years; only the taxa meeting the LDA significance threshold > 2 are shown. **(C)** LEfSe analysis of differentially abundant taxa of endophytic bacteria in *A. euchroma* grown for different years; taxa are listed on the right side of the pedigree plot. **(D)** Bar chart showing LDA scores for endophytic bacterial taxa in root samples of *A. euchroma* cultivated for different years; only the taxa meeting the LDA significance threshold > 2 are shown.

The LEfSe technique [57] was used to identify differentially abundant taxa in *A. euchroma* cultivated for different years. The class diagrams generated by LEfSe showed the differences between the four organs at the phylum, class, order, family, and genus levels. Each successive circle represents a phylogenetic level. The colored areas indicate the richness of taxa in different organs. Statistically different biomarkers at the genus level in endophytic fungi were found by the LEfSe analysis. Fig 5A shows that 13 fungi species were significantly enriched in the roots of the X-1Z treatment. 2 species of fungi were significantly enriched in X-2Z roots. 6 species of fungi were significantly enriched in the X-3Z roots. In addition, in roots of the X-4Z treatment, 10 species of fungi were significantly enriched. As observed in Fig 5B, the LDA analysis (LDA > 2) showed that the significantly different microflora in the X-1Z roots was reduced, with only 10 species. Among them, *Nectriaceae* had the greatest effect on 1-year-old *A. euchroma*, and both fungi exerted significant effects on 2-year-old *A. euchroma*. Furthermore, the emergence of four unclassified *Basidiomycota* species exerted the greatest effect on 3-year-old *A. euchroma*. *Cadophora* had the maximum effect on 4-year-old *A. euchroma*.

Statistically different biomarkers were found in endophytic bacterial communities at the genus level. As shown in Fig 5C, the LEfSe analysis revealed that 4 bacterial species were significantly enriched in X-1Z roots. 16 bacterial species were significantly enriched in X-2Z roots. In the X-3Z group, no endophytic bacterial taxa significantly affected species differences. And 2 bacteria species were significantly enriched in X-4Z roots. The LDA analysis (LDA > 2) in Fig 5D showed that *g_unclassified_Sphingomonadaceae* appeared in the X-1Z roots and had a significant effect on the formation of 1-year-old cultivated *A. euchroma*. However, p_Proteobacteria still exerted the greatest overall influence. The significantly different microflora in the X-2Z roots decreased drastically, leaving only 10 species. p_Bacteroidota and *c_Bacteroidia* had the maximum effect on 2-year-old *A. euchroma*. 3-year-old *A. euchroma* was not significantly affected by any endophytic bacterial taxa. In addition, the maximum effect on 4-year-old *A. euchroma* was caused by f_Xanthobacteraceae and *g_Tardiphaga*.

### Correlation analysis of endophytes and metabolites in *A. euchroma*

**Fig 6:**
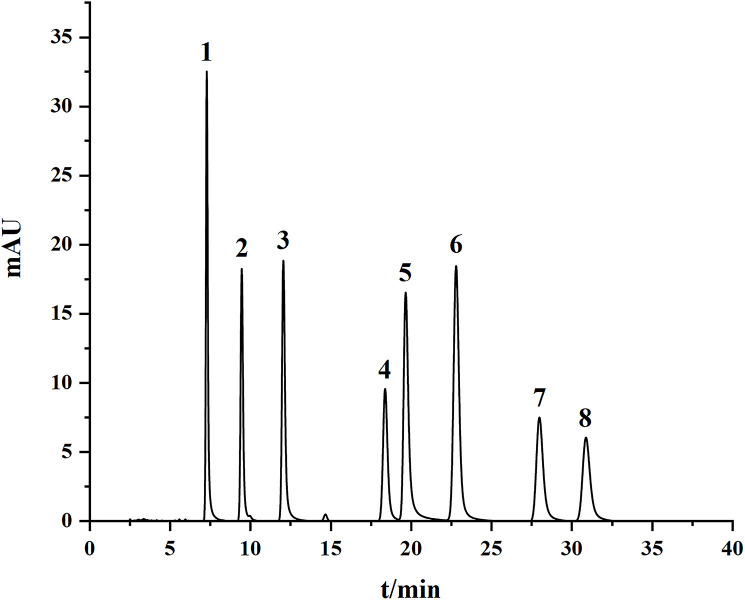
HPLC chromatograms of metabolite standards in *A.euchroma*; 1: Alkannin 2: *β*-Hydroxyisovalerylshikonin; 3: Acetylshikonin; 4: *β*-Acetoxyisovaleryshikonin; 5: Deoxyshikonin; 6: Isobutyrylshikonin; 7: *β,* *β*-Dimethylacrylshikonin; 8: Isovalerylshikonin.

**Fig 7:**
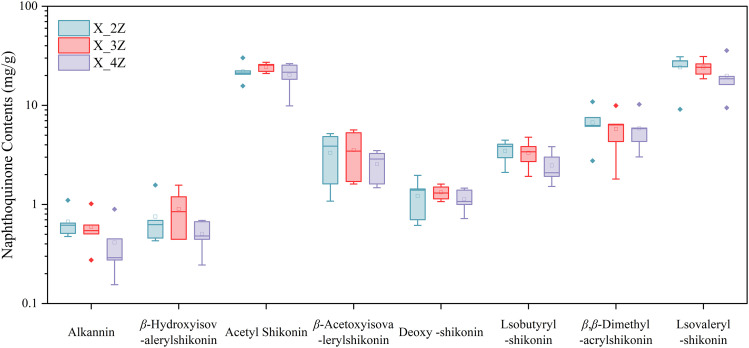
Boxplot of the contents of eight naphthoquinone components in 2-4-year-old *A. euchroma.*

**Fig 8:**
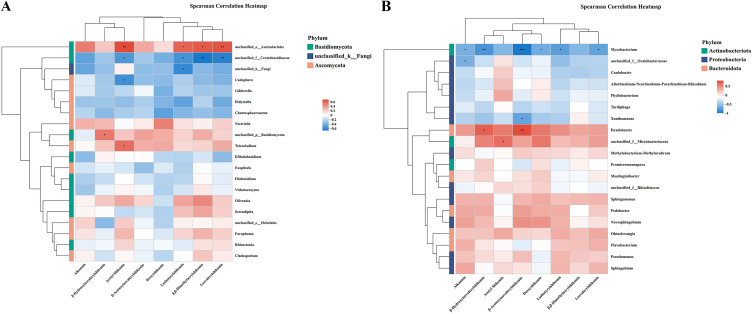
Heat map of the correlation between (A) endophytic fungi and (B) endophytic bacteria and secondary metabolites in *A. euchroma* cultivated for different years. *: *P ≤ 0.05*,**: *P ≤ 0.01* and ***: *P ≤ 0.001.*

Spearman’s correlation heat map was used to analyze the correlation between the top 20 ASV genera of endophytes and eight naphthoquinone chemical compounds in the root of *A. euchroma* across 1–4 cultivation years. Fig 6 and Fig S1 present the HPLC chromatograms of the reference standards and the 2–4-year-old samples for the eight naphthoquinone components in *A. euchroma*, indicating that these metabolites can be efficiently detected using HPLC technology. The regression equations and R² values for the reference standards of the eight naphthoquinone components are presented in Table S1. The contents of the eight naphthoquinone components in *A. euchroma* from different growth years are shown in Fig 7 and S2 Table. Fig 8A shows that among the taxa of endophytic fungi, *unclassified_o_Auriculariales* was positively correlated (*P ≤ 0.05*) with acetylshikonin, isobutyrylshikonin, *β, β*-dimethylacrylshikonin, and lsovaleryshikonin. Moreover, the effects on acetylshikonin and isovalerylshikonin were also significant at the 0.01 level (*P ≤ 0.01*), and unclassified_p__Basidiomycota was positively correlated with *β*-hydroxyisovalerylshikonin (*P ≤ 0.05*). Similarly, *Tetracladium* was positively correlated with acetylshikonin (*P ≤ 0.05*), while *unclassified_f_Ceratobasidiaceae* was negatively correlated with acetylshikonin, isobutyrylshikonin, *β, β*-dimethylacrylshikonin, and isovalerylshikonin (*P ≤ 0.05*), with the last two also showing significant correlations at the 0.01 level (*P ≤ 0.01*). In addition, *Cadophora* was negatively correlated with isobutyrylshikonin and acetylshikonin, respectively (*P ≤ 0.05*).

As shown in Fig 8B, among the endophytic bacteria, *Mycobacterium* was negatively correlated with alkannin, deoxyshikonin, isobutyrylshikonin, and isovalerylshikonin (*P* *≤ 0.05*).It showed a significant negative correlation with *β*-hydroxyisovalerylshikonin (*P ≤ 0.01*) and an extremely significant negative correlation with *β*-acetoxyisovalerylshikonin (*P ≤ 0.001*). *Xanthomonas* was negatively correlated with *β*-acetoxyisovalerylshikonin (*P* *≤ 0.05*). In contrast, *Dyadobacter* was positively correlated with both *β*-hydroxyisovalerylshikonin and *β*-acetoxyisovalerylshikonin (*P ≤ 0.05*), and the correlation was more significant (*P ≤ 0.01*) with the latter.

### Functional Prediction of endophytes in A. euchroma by FUNGUILD and PICRUSt2 analyses

**Fig 9:**
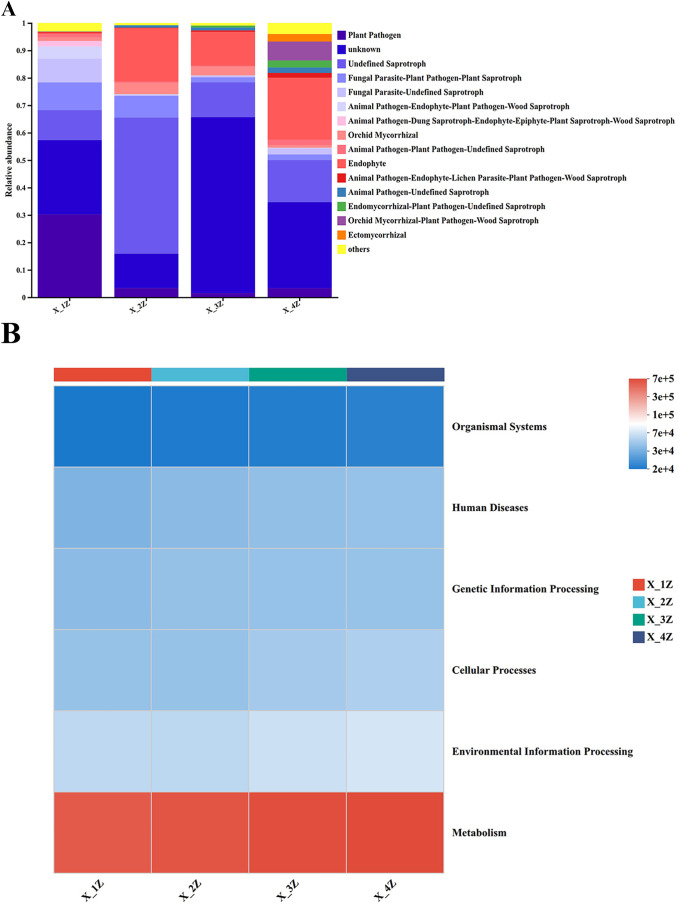
Functional prediction of the endophytic fungi and bacteria in *A. euchroma* cultivated for 1–4 years; (A) Plot of relative abundance of the predicted nutrient types of endophytic fungi (B) Heatmap of the predicted KEEG functional pathways (KEEG level 1) of endophytic bacterial communities.

Fungi Functional Guild (FUNGuild) is a tool used for the classification and analysis of fungal communities in microecological guilds [58,59]. Analysis using FUNGuild software revealed a total of 15 major eco-functional taxa of endophytes in cultivated *A. euchroma* annotated with a combined species abundance threshold of < 0.01, along with unknown functional taxa (Fig 9A). The trophic modes of endophytic fungi were mainly the two major groups: saprotrophs and pathotrophs. Additionally, there were four types of symbiotrophs, including endophytes, orchid mycorrhiza, ectomycorrhiza, and endomycorrhiza, as well as nine mixed trophic types. A large proportion of colonies belonged to species with unknown functions, which require further investigation. Bacterial functional prediction was performed using PICRUSt2; six major categories of metabolic pathways at the primary functional level were obtained from the KEGG database, including metabolism, genetic information processing, environmental information processing, cellular processes, organismal systems, and human diseases. Metabolic pathways were the major components in all samples (Fig 9B).

## Discussion

With the development and maturity of novel sequencing technologies, the study of microbial diversity and community structure has become increasingly intensive. HTS can compensate for the limitations of selection made by traditional culture methods, allow for the in situ analysis of microbial population structure, and provide information on microbial community structure most similar to that in plants under natural conditions. In addition, we found that the classification of a large number of endophytic fungi could not be possible. Indeed, only about 10,000 out of approximately 5.1 million fungal species have been classified and characterized [60]. Therefore, HTS is more effective in comprehensively exploring the structural composition of endophytic fungi and inter-root soil microbial communities than traditional methods used for the isolation of colonies from microbial cultures [61]. In this study, we performed the analysis of diversity, species abundance, endophytic markers, and functional prediction of endophytic fungi and bacteria in *A. euchroma* cultivated for 1–4 years using high-throughput sequencing.

HTS revealed that the endophytic fungal ASVs in cultivated *A. euchroma* were placed within 12 phyla, 32 classes, 76 orders, 153 families, 259 genera, and 367 species. The endophytic bacterial ASVs in cultivated *A. euchroma*, however, were distributed within 18 phyla, 37 classes, 89 orders, 139 families, 226 genera, and 1228 species. In the Venn diagram, the number of fungal ASVs in *A. euchroma* grown for 1–4 years was in the following order: X-4Z (291) ＞X-1Z (249) ＞X-3Z (175) ＞X-2Z (141), with only 81 shared fungal ASVs. The number of bacterial ASVs was in the order of X-2Z (171) ＞X-3Z (136) ＞X-4Z (126) ＞X-1Z (125), with only 16 bacterial ASVs shared by all treatment groups. This indicates that the number of endophytic fungal ASVs was much larger than that of endophytic bacterial ASVs in roots of cultivated *A. euchroma*, with the number of endophytic fungi increasing, while that of bacteria tending to decrease every year with plant aging, which needs to be further investigated. Numerous studies have shown that plant roots are the colonization sites for endophyte communities [62]. Plant roots can release large amounts of photosynthates or exudates, which can influence the root tissue microbial communities [63,64]. Root secretions including organic acids, amino acids, and proteins may be involved in the recruitment of bacterial endophytes that reside in the root tissue. Further analysis by Wang et al [52] showed a high diversity of root endophytic fungi, which may be due to the presence of high amounts of apoplastic materials (fallen leaves, twigs, and dead roots) and root secretions at the root-soil interface. They can provide sufficient carbon, nitrogen, and trace elements to fungi inhabiting the root tissue, thus, contributing to their abundance. There are both mutual enhancement and mutual inhibition among the bacterial and fungal taxa, and the entire microbial community is relatively balanced. Similarly, in this study, we found that the dominant fungal phylum was Ascomycota, whereas Proteobacteria was the dominant bacterial phylum. As a dominant fungal phylum in most plants, Ascomycota is mainly found in the roots of plants such as Pinus thunbergii and Gentiana. Proteobacteria are a large family of bacteria that play an important role in biological nitrogen fixation in some herbaceous plants like *Panax quinquefolius L.*[39] and are used to synthesize nitrogen-containing substances such as alkaloids and proteins. The Venn diagram of ASVs showed a decreasing trend in unique ASV numbers of bacteria with every passing year. This is consistent with the results obtained by Pan et al [65] on the diversity of inter-root microbial communities in apples grown for different years and by Miguel et al [66] on the diversity of endophytic bacterial communities in *eucalyptus* at different growth stages. The age of the plant had reportedly an effect on nutrients in the plant itself so that the micro-organisms in the host plant were less abundant. Among bacteria, Proteobacteria and Actinobacteria are mainly involved in plant nitrogen fixation and the decomposition of plant organic residues, respectively [67,68]. These processes, in turn, make the organic matter mainly accumulated in plant roots and thus unable to be absorbed in the short term, which leads to the increase or decrease in the relative abundance of such bacterial genera. For example, Zhang et al [69] demonstrated that as plants aged, the number of endophytic bacteria in the cultivated ginseng decreased and a stable colony was formed, which promoted the accumulation of effective medicinal components in the plant. This finding also corroborates the phylum-level relative abundance of endophytic bacteria in 1–4-year-old cultivated *A. euchroma* (Fig 3C).

The relative abundance of Ascomycota and Basidiomycota was higher among the fungi, and Ascomycota and Basidiomycota were the major decomposer communities in the soil [70]. Ascomycota is mostly saprophytic and can decompose hard-to-degrade organic matter; Basidiomycota is mostly saprophytic or parasitic and decomposes lignocellulose in moist soils [71]. The root-associated fungal community structure in cultivated *A. euchroma* has dynamic changed with planting age. The natural succession of fungi in *A. euchroma* roots was similar to that in *Fagus longipetiolata* [72], which may be attributed to the fact that environmental components such as soil in cultivated *A. euchroma* gradually changed with cultivation age, resulting in differences in the composition and structure of the fungal community [73]. Our conclusion stating that the endophyte community richness was greater for fungi than for bacteria is consistent with the finding obtained by the analysis of endophyte diversity in the *Dendrobium* root system performed by Liu Fangzhou et al [36]. In the above study, endophytic fungi were more likely to vary in their community richness under different conditions, compared to endophytic bacteria. In conclusion, different climates, geographic environments, and relative abundances all caused differences in the number of endophytic fungal and bacterial communities present in cultivated *A. euchroma* [74]. Although this study preliminarily established a correlation between endophytes and shikonin accumulation in *A. euchroma*, a limitation lies in the absence of data on rhizosphere soil physicochemical properties. These factors are key determinants influencing both the microbial community and the accumulation of secondary metabolites in *A. euchroma*. Building on these findings, subsequent research will focus on characterizing these soil parameters and the rhizosphere microbiome. This approach will facilitate a more comprehensive understanding of the complex interplay among soil conditions, microbial communities, and the quality of *A. euchroma*.

Many reports have confirmed that endophytic bacteria can produce biologically active secondary metabolites in plants. In this study, we investigated the correlation between the diversity of endophytes and the contents of eight secondary metabolites in the inter-root zone of *A. euchroma* cultivated for 1–4 years using heatmaps. Spearman’s correlation analysis showed that *β*-hydroxyisovalerylshikonin, acetylshikonin, isobutyrylshikonin, *β,β*-dimethylacrylshikonin, and isovalerylshikonin were correlated with endophytic fungal diversity and abundance (*P* ≤ 0.05). This finding is consistent with the results achieved by Chen et al [38] and Cui et al [25] who reported significant correlations between metabolites and endophytic fungi in *Rheum palmatum* and *Cynomorium songaricum Rupr*. *β*-hydroxyisovalerylshikonin, acetylshikonin and *β*-acetoxyisovalerylshikonin were positively correlated (*P* ≤ 0.05) with the diversity and abundance of endophytic bacteria, which is in line with the results obtained by Huo [75] and Xue [76] on the correlation between metabolites and endophytic bacteria in *Khaya A. Juss* and *Ginkgo biloba L*.

According to the 2020 edition of the *Chinese Pharmacopoeia*, not less than 0.30% of *β, β*-dimethylacrylshikonin (C_21_H_22_O_6_) detected by the HPLC method is required for the production of high-quality *A. euchroma*. Therefore, in the correlation analysis between endophytes and secondary metabolites in cultivated *A. euchroma* from different years (Fig 7), we will focus on the representative endophytic taxa that influence the content of *β,β*-dimethylacrylshlkannin. First, *unclassified_o__Auriculariales* showed a positive correlation with its content (P ≤ 0.05). *Auriculariales* is an order of fungi within the phylum *Basidiomycota*, most members of which are saprotrophs. Although no clear evidence in the literature suggests that *Auriculariales* have growth-promoting effects per se, studies on *Auricularia* mushrooms have revealed that these fungal strains harbor terpene synthase gene clusters [77]. This finding provides genomic evidence that *Auricularia*-like fungi possess the genetic basis for synthesizing complex secondary metabolites. Therefore, we hypothesize that *unclassified_o__Auriculariales*, colonizing the roots of cultivated *A. euchroma*, may also activate key enzyme genes involved in the biosynthetic pathway of terpenoids such as *β,β*-dimethylacrylshikonin through a similar fungal elicitor mechanism, thereby promoting the accumulation of shikonin derivatives and resulting in a positive correlation with their content. In contrast, *unclassified_f__Ceratobasidiaceae* showed a significant negative correlation with its content (*P ≤ 0.01*). As an important member of the phylum *Basidiomycota*, the family *Ceratobasidiaceae* includes several destructive plant pathogens. Studies have shown that in plant–pathogen interactions, plants typically synthesize and accumulate secondary metabolites (e.g., tannins) as defensive weapons to inhibit pathogen infection and growth [78]. Therefore, when a negative correlation is observed, it suggests that *Ceratobasidiaceae*, as a pathogen, exists in high abundance, and its infection process may interfere with or inhibit the biosynthetic pathway of shikonin derivatives in cultivated *A. euchroma*, thereby leading to a decrease in their content. In addition, we found that *Mycobacterium* showed an extremely significant negative correlation with *β*-acetoxyisovalerylshikonin (*P ≤ 0.001*). Some species of *Mycobacterium* are pathogenic or opportunistic pathogens. Numerous studies have shown that plant-derived secondary metabolites, such as flavonoids, quinones, and terpenoids, exhibit significant inhibitory effects on *Mycobacterium* [79]. From this, we can infer that when the abundance of *Mycobacterium* is high, it may compete for nutrients or interfere with metabolic pathways, resulting in reduced resources for the synthesis of secondary metabolites in plants. *Dyadobacter* showed a significant positive correlation with *β*-acetoxyisovalerylshikonin (*P ≤ 0.01*). As an important plant growth-promoting rhizobacterium (PGPR), it has been confirmed to colonize plant tissues as an endophyte [80]. This preliminary result showed that the endophytes in the roots of *A. euchroma* were closely related to the contents of secondary metabolites, and the secondary metabolites involved in different biosynthetic pathways were also significantly (both positively and negatively) correlated with the endophytes. This finding confirms that endophytes may be involved in the accumulation of secondary metabolites in *A. euchroma* plants, which deserves further investigation. The next step is to place more emphasis on isolating endophytic bacteria from *A. euchroma* plants and inoculating them into the same plants to verify their roles in the accumulation of secondary metabolites and the mechanisms controlling it.

In this study, FUNGuild was used to predict the functions of endophytic fungi in cultivated *A. euchroma*. The results showed that the functions of endophytic fungi in *A. euchroma* were primarily composed of two dominant trophic modes: saprotrophs and pathotrophs. However, this study still has certain limitations, necessitating further classification and prediction of rhizosphere soil fungi to fully explore the role of fungal diversity. PICRUSt2 is a reliable tool for predicting the metabolic functions of bacterial communities [81,82]. The primary function of all endophytic bacteria in *A. euchroma* was metabolism, which is consistent with the findings of Pii et al. [83] regarding bacterial function prediction in barley and tomato roots. Nevertheless, the utility of PICRUSt2 remains limited, as it can only predict the functions of the bacteria involved but cannot elucidate the underlying mechanisms of their effects. In the future, methods such as metagenomics could be employed for more in-depth studies to better understand the functions of endophytic bacteria in *A. euchroma*.

## Conclusions

The results of the present study showed that a total of 1161 ASVs of endophytic fungi, belonging to 12 phyla, 32 classes, 76 orders, 153 families, 259 genera, and 367 species, were detected in the root tissue of *A. euchroma* cultivated for 1–4 years old. Moreover, 673 ASVs of endophytic bacteria, belonging to 18 phyla, 37 classes, 89 orders, 139 families, 226 genera, and 267 species, were detected. The dominant group among endophytic fungi was *Ascomycota*, while among endophytic bacteria, the dominant group was *Proteobacteria*. Spearman’s correlation analysis showed that *β*-hydroxyisovalerylshikonin, acetylshikonin, isobutyrylshikonin, *β,β*-dimethylacrylshikonin, and isovalerylshikonin were correlated with endophytic fungal diversity and abundance (*P ≤ 0.05*). Endophytic fungi in *A. euchroma* had 15 major ecofunctional taxa, of which saprotrophic bacteria were utterly dominant, followed by the nutritional types of bacteria, while symbiotic bacteria were the least dominant bacteria. Endophytic bacteria, on the other hand, had metabolism as their main function.

## Supporting information

S1 Fig HPLC chromatograms of 2-year-old samples (A), 3-year-old samples (B), and 4-year-old samples (C). (DOCX)

S1 Table Regression equations and linear ranges of eight naphthoquinone reference standards. (XLSX)

S2 Table Contents of eight naphthoquinone components in 2–4-year samples (mg/g). (XLSX)
