# Supplementary figures and images for: Endophyte diversity and its correlation with naphthoquinone metabolites in cultivated Arnebia euchroma across different growth years
Source: PLoS One. 2026 May 21;21(5):e0348171. doi: 10.1371/journal.pone.0348171 (PMC13193543; doi:10.1371/journal.pone.0348171)

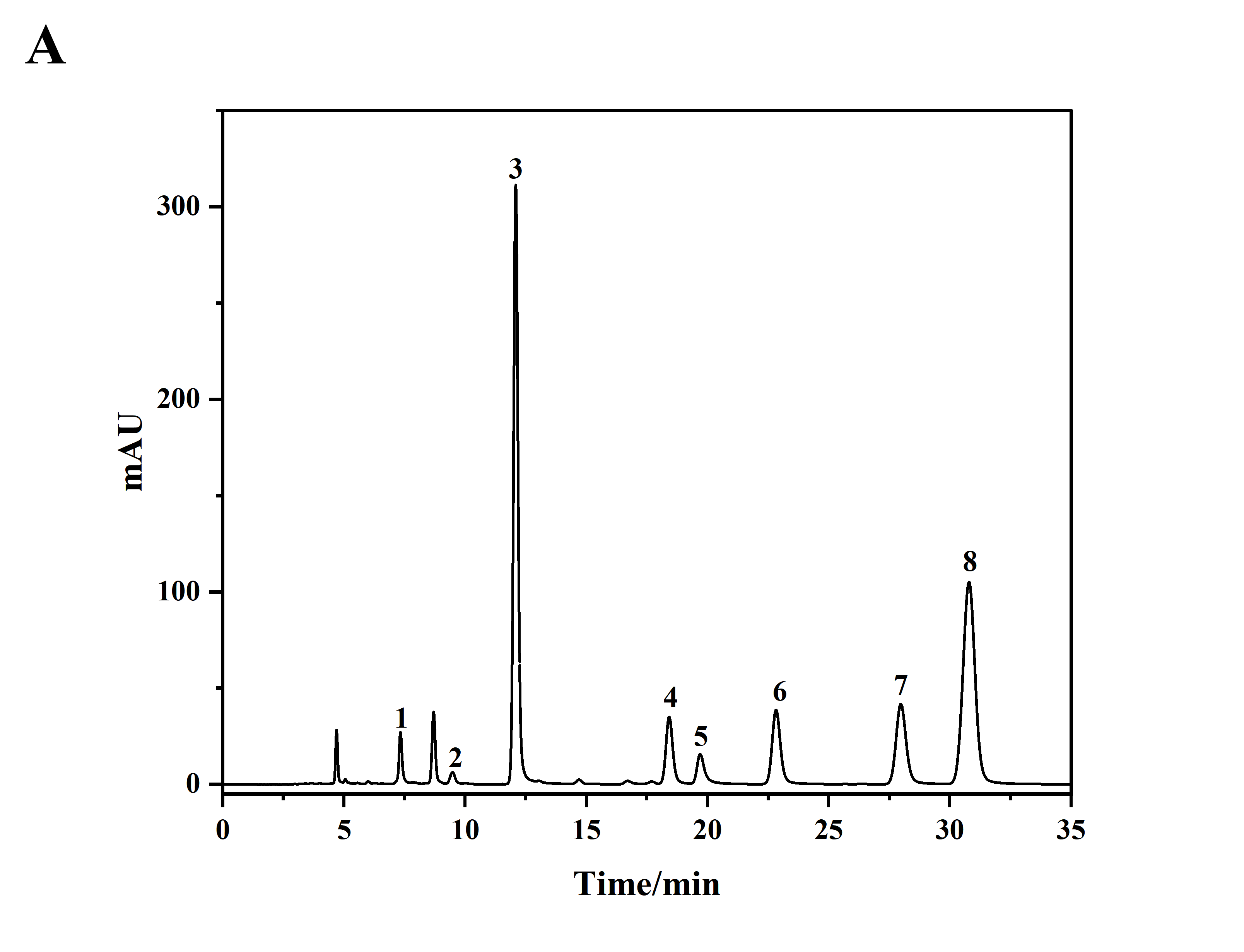

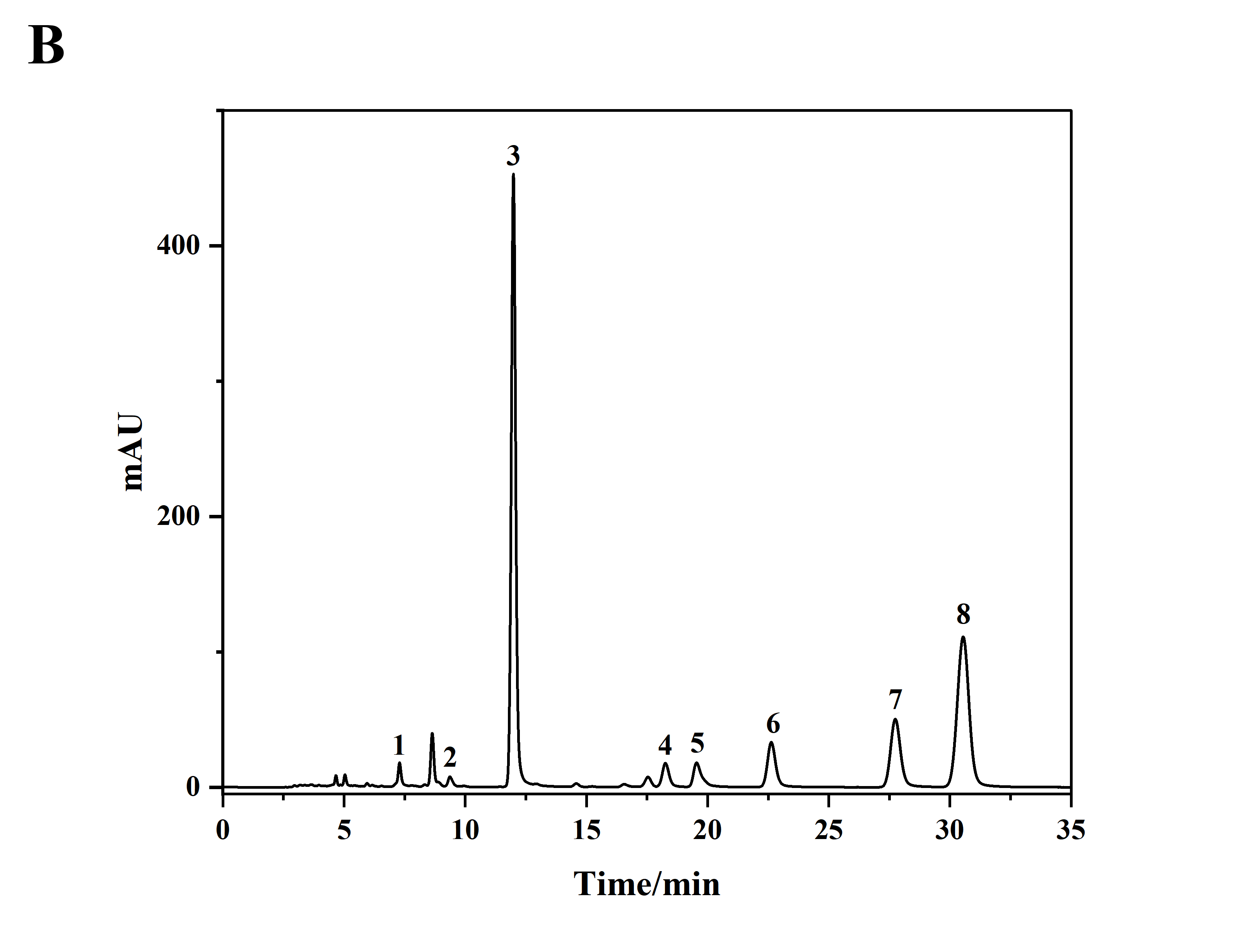

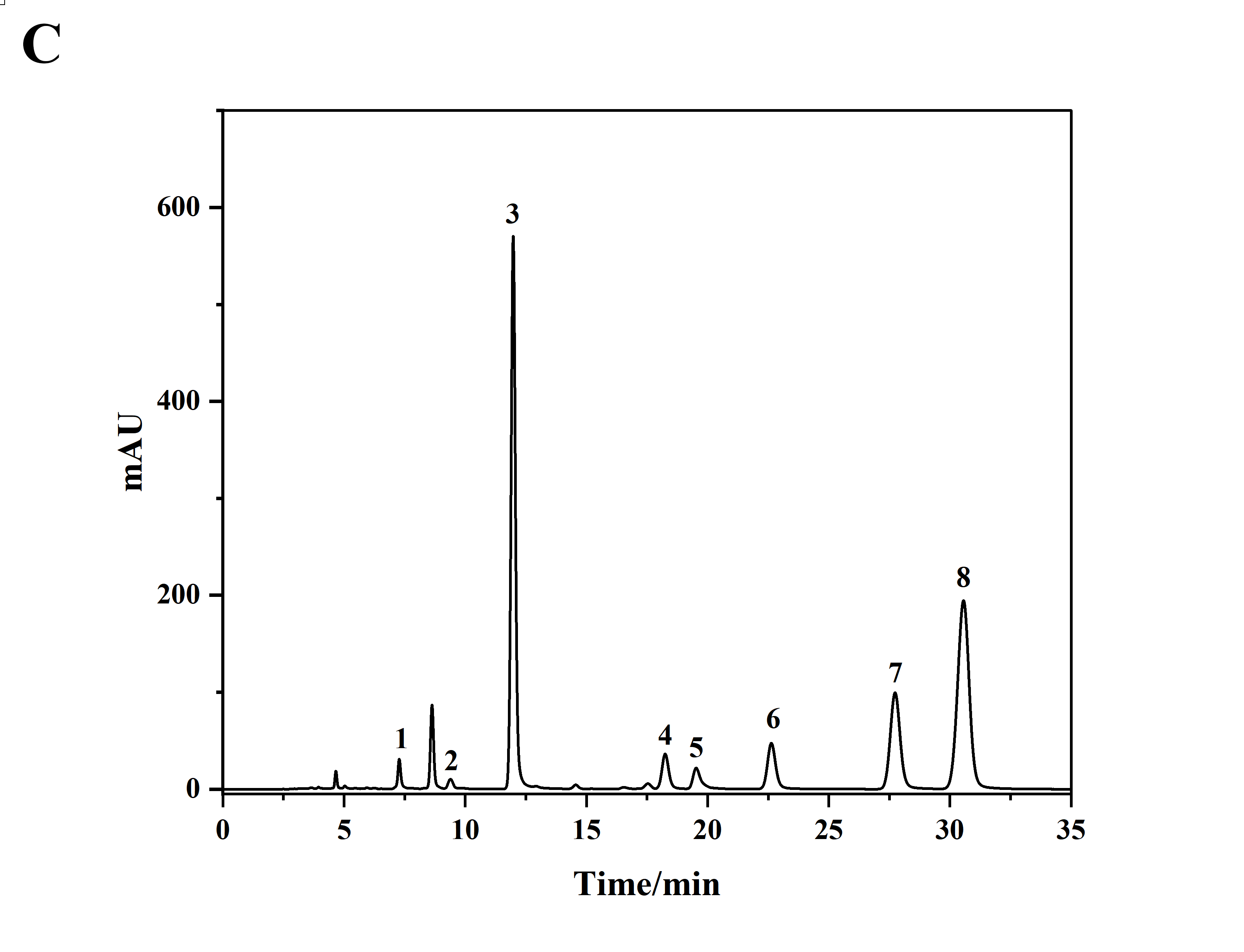


**Figure S1.** HPLC chromatograms of 2-year-old samples (**A**), 3-year-old samples (**B**), and 4-year-old samples (**C**).

Supplement: S1 Fig — (DOCX) [file pone.0348171.s001.docx]
